# 

**Published:** 1812-10

**Authors:** 


### MONTHLY CATALOGUE OF MEDICAL BOOKS.

ELEMENTS of Physiology. By A. Richerand, Professor of the Faculty of Medicine of Paris, &c. &c. Translated from the French by G. J. M. de Lys, M.D. Member of the Royal College of Surgeons in London. 8vo.—Underwood.

Underwood's Catalogue of Medical Books for 1812-13, new and second-hand, containing a large assortment of French books.

Gregory's Conspectus Medicinæ. New edition. 8vo.—Underwood.

Novum Nosologiæ Methodicæ Systema, Auctore F. Swediana, M. D. 8vo.—Underwood.

A Treatise on the Diseases of Arteries and Veins, comprising the Treatment of Aneurism and Wounded Arteries. By James Hodgson, Member of the Royal College of Surgeons in London.—Underwood.

---

### *NOTICES TO CORRESPONDENTS.*

*We regret that expressions, calculated to excite uneasiness in the minds of some of our correspondents, have escaped correction in two or three of our late articles. It is very far from our desire to encourage undue warmth in discussing medical opinions, much less is it our wish to foment animosity between individuals. The public at large is no ways interested in local dissention, or personal enmity; we therefore trust that, in future, gentlemen who honor us with their communications, will not compel us to reject them on account of unbecoming language: especially, we deem it unjust for anonymous writers to attack with virulence, and abuse, characters of known respectability. We emulate free and liberal discussion, but will never knowingly be instrumental in degrading the medical character.*

*Articles will be inserted in due course from Mr. Woodham; Mr. Davies; Mr. Tytler; an impartial Medical Man; a practical Physician; L——, surgeon; &c. &c.*

